# A gut microbial metabolite of linoleic acid ameliorates liver fibrosis by inhibiting TGF-β signaling in hepatic stellate cells

**DOI:** 10.1038/s41598-023-46404-5

**Published:** 2023-11-03

**Authors:** Nanaho Kasahara, Yukiko Imi, Reina Amano, Masakazu Shinohara, Kumiko Okada, Yusei Hosokawa, Makoto Imamori, Chiaki Tomimoto, Jun Kunisawa, Shigenobu Kishino, Jun Ogawa, Wataru Ogawa, Tetsuya Hosooka

**Affiliations:** 1 Laboratory of Nutritional Physiology, Graduate School of Integrated Pharmaceutical and Nutritional Sciences, University of Shizuoka, 52-1 Yada, Suruga-Ku, Shizuoka, 422-8526 Japan; 2 Division of Molecular Epidemiology, Department of Future Medicine Sciences, Kobe University Graduate School of Medicine, Kobe, Hyogo 650-0017 Japan; 3 The Integrated Center for Mass Spectrometry, Kobe University Graduate School of Medicine, Kobe, Hyogo 650-0017 Japan; 4 Division of Diabetes and Endocrinology, Department of Internal Medicine, Kobe University Graduate School of Medicine, Kobe, Hyogo 650-0017 Japan; 5 Noster Inc., Muko-Shi, Kyoto, 617-0006 Japan; 6 Laboratory of Vaccine Materials and Laboratory of Gut Environmental System, Microbial Research Center for Health and Medicine, National Institutes of Biomedical Innovation, Health, and Nutrition (NIBIOHN), Osaka, 567-0085 Japan; 7 Division of Applied Life Sciences, Graduate School of Agriculture, Kyoto University, Sakyo-Ku, Kyoto, 606-8502 Japan

**Keywords:** Molecular medicine, Preclinical research

## Abstract

The antidiabetic drug pioglitazone ameliorates insulin resistance by activating the transcription factor PPARγ. In addition to its blood glucose–lowering action, pioglitazone exerts pleiotropic effects including amelioration of nonalcoholic fatty liver disease (NAFLD)/nonalcoholic steatohepatitis (NASH). The mechanism by which pioglitazone achieves this latter effect has remained unclear, however. We here show that pioglitazone administration increases the amount of linoleic acid (LA) metabolites in adipose tissue of KK-Ay mice. These metabolites are produced by lactic acid bacteria in the gut, and pioglitazone also increased the fraction of *Lactobacillus* in the gut microbiota. Administration of the LA metabolite HYA (10-hydroxy-cis-12-octadecenoic acid) to C57BL/6 J mice fed a high-fat diet improved liver histology including steatosis, inflammatory cell infiltration, and fibrosis. Gene ontology analysis of RNA-sequencing data for the liver revealed that the top category for genes downregulated by HYA treatment was related to extracellular matrix, and the expression of individual genes related to fibrosis was confirmed to be attenuated by HYA treatment. Mechanistically, HYA suppressed TGF-β–induced Smad3 phosphorylation and fibrosis-related gene expression in human hepatic stellate cells (LX-2). Our results implicate LA metabolites in the mechanism by which pioglitazone ameliorates liver fibrosis, and they suggest that HYA is a potential therapeutic for NAFLD/NASH.

## Introduction

Nonalcoholic fatty liver disease (NAFLD) is the most prevalent form of chronic liver disease in industrialized countries, affecting one-fourth of the global population^1–3^. NAFLD is characterized by excessive fat accumulation in the liver related to obesity and insulin resistance, and it is associated with a high rate of type 2 diabetes, dyslipidemia, and hypertension^4–6^. It ranges in severity from simple steatosis to nonalcoholic steatohepatitis (NASH), which is estimated to account for 10% to 20% of NAFLD cases, and it can progress to cirrhosis and hepatocellular carcinoma^7,8^. Although multiple factors—including diet, genetic variants such as those of *PNPLA3*, insulin resistance, oxidative stress, excessive production of proinflammatory adipokines, chronic inflammation, and gut dysbiosis—have been implicated in the development of NAFLD/NASH^9–11^, the pathology of this condition is complex and not fully understood. There are currently no approved medications for NAFLD/NASH.

Histological features of NASH include steatosis, inflammatory cell infiltration, hepatocyte ballooning, and fibrosis, the latter of which is the strongest predictor of liver-related events and long-term mortality^12,13^. Intervention to target liver fibrosis may therefore provide new therapeutics for NAFLD/NASH. Various factors and pathways have also been implicated in the pathogenesis of liver fibrosis associated with NAFLD/NASH. Activation of hepatic stellate cells and the consequent overproduction of collagen plays a central role in the development of liver fibrosis^14^, with transforming growth factor–β (TGF-β) being thought to be a key fibrogenic factor that activates hepatic stellate cells through a Smad3-dependent signaling pathway^15^.

Pioglitazone is widely administered for the treatment of type 2 diabetes. This antidiabetic agent exerts an insulin-sensitizing effect by binding to and activating the transcription factor PPARγ (peroxisome proliferator–activated receptor γ)^16,17^. In addition to its lowering of blood glucose concentration, pioglitazone exerts pleiotropic effects including amelioration of NAFLD/NASH^18–20^. Several randomized controlled trials of pioglitazone for the treatment of NAFLD/NASH revealed that, despite the fact that the dose and duration of pioglitazone administration as well as the presence of diabetic complications in the trial subjects differed among the studies, pioglitazone significantly ameliorated liver histology including steatosis, hepatocyte ballooning, and inflammatory cell infiltration compared with placebo^21–27^. Furthermore, some studies have shown that pioglitazone ameliorates liver fibrosis^22,24–26^. The mechanisms by which pioglitazone improves these pathological conditions have remained unclear, however.

We previously showed that linoleic acid (LA), which is widely present in edible fats and oils, is saturated by lactic acid bacteria represented by *Lactobacillus* through a novel metabolic process and that LA metabolites—including hydroxy fatty acids (hydrated fatty acids), oxo fatty acids, and conjugated fatty acids—are produced as intermediates in this process^28^. Given that the amount of LA metabolites is extremely low in germ-free mice compared with specific pathogen–free mice, these metabolites are thought to be produced by gut microorganisms such as lactic acid bacteria represented by *Lactobacillus* in the gut^28^.

In the present study, we performed lipidomics analysis in genetically obese and diabetic KK-Ay mice in order to explore the mechanism by which pioglitazone ameliorates NAFLD/NASH. We found that pioglitazone treatment increased the amount of LA metabolites in adipose tissue of these mice, and that this effect was accompanied by an increase in the fraction of *Lactobacillus* in the gut microbiota. We therefore also investigated the effect of 10-hydroxy-*cis*-12-octadecenoic acid (HYA), an LA metabolite, on the development of NAFLD/NASH in a diet-induced mouse model of this condition.

## Materials and methods

### Animal studies

Male 7-week-old KK-Ay mice were obtained from CLEA Japan (Tokyo, Japan), housed individually, fed a normal diet (CE-2, CLEA Japan), and acclimatized for 1 week. They were then divided into two groups and fed for 4 weeks with either a control diet (CE-2) or the same diet supplemented with 0.01% pioglitazone (Fujifilm Wako Pure Chemical, Osaka, Japan). Male 4-week-old C57BL/6 J mice obtained from CLEA Japan were similarly acclimatized for 1 week and then divided into four groups that were fed for 26 weeks with either a normal diet (CE-2) or a high-fat diet (HFD32, CLEA Japan) that was supplemented or not with 1% HYA or 1% LA (Noster Inc., Kyoto, Japan). All mice were housed at 21° to 25 °C and maintained on a 12-h-light, 12-h-dark cycle in the animal facility at Kobe University Graduate School of Medicine or University of Shizuoka. All animal experiments were approved by and performed in accordance with the guidelines of ARRIVE, the animal ethics committees of University of Shizuoka (permission no. 215326) and Kobe University Graduate School of Medicine (permission no. P171006). Mice were anaesthetized by isofurane (VIATRIS, Tokyo, Japan) at a flow rate of 1.5 L/min at 2–3% concentration as administered by an animal anesthetizer (SN-487-0 T Air; SHINANO manufacturing, Tokyo, Japan), and then quickly euthanized by cervical dislocation to collect tissues.

### Cell culture and treatment

LX-2 human hepatic stellate cells (Sigma Aldrich, St. Louis, MO, USA) were maintained under a humidified atmosphere of 5% CO_2_ at 37 °C in Dulbecco’s modified Eagle’s medium (DMEM) supplemented with 2% fetal bovine serum (NICHIREI, Tokyo, Japan), penicillin (100 U/mL), and streptomycin (100 μg/mL). The cells were transferred to 60-mm dishes and cultured to 80% to 90% confluence before experiments.

### LC–MS/MS analysis

Deuterated internal standards (d4-leukotriene B_4_, d8-5-hydroxyeicosatetraenoic acid, d4-prostaglandin E_2_, and d5-resolvin D2) representing each chromatographic region of identified lipid mediators were added (500 pg each) to samples to facilitate quantification. Samples were subjected to solid-phase extraction on a C_18_ column as described previously^29^ and were then analyzed by liquid chromatography (LC) and tandem mass spectrometry (MS/MS) with a Qtrap 6500 instrument (Sciex, Framingham, Massachusetts, USA) equipped with an LC-30AD high-performance LC system (Shimadzu, Kyoto, Japan). A Zorbax Eclipse Plus C_18_ column (100 by 4.6 mm, 3.5 mm; Agilent Technologies, Santa Clara, CA, USA) was subjected to elution with a gradient of methanol/water/acetic acid (v/v/v) from 55:45:0.01 to 98:2:0.01 at a flow rate of 0.4 mL/min. For monitoring and quantification of the targeted lipid mediators, a multiple reaction monitoring (MRM) method was developed with signature ion pairs Q1 (parent ion)/Q3 (characteristic fragment ion) for each molecule. Identification of each lipid mediator was based on published criteria for LC retention time, specific fragmentation pattern, and at least six diagnostic fragmentation ions. Quantification was performed on the basis of peak area on the MRM chromatograph, with linear calibration curves being obtained with authentic standards for each compound. Under these conditions, HYA and 10-hydroxy-*trans*-11-octadecenoic acid (HYC) were not clearly separated and expressed as HYA + HYC.

### Analysis of fecal microbiota

Fecal samples were collected at 3 weeks after the onset of pioglitazone treatment and stored at  − 80 °C until analysis. Extraction of DNA, 16S rRNA gene amplification, and sequencing with the MiSeq system (Illumina, San Diego, CA, USA) were performed by Bioengineering Lab. Co. (Kanagawa, Japan).

### Blood parameters

Blood glucose concentration was measured with a Glutest kit (Sanwa Kagaku Kenkyusho, Nagoya, Japan), plasma insulin concentration with a Mouse Insulin ELISA kit (Mercodia AB, Uppsala, Sweden), and plasma aspartate aminotransferase (AST) and alanine aminotransferase (ALT), triglyceride, cholesterol, and nonesterified fatty acid (NEFA) levels with Transaminase CII, Triglyceride E, Cholesterol E, and NEFA C test kits (Fujifilm Wako Pure Chemical).

### Histological analysis

Serial sections of the liver were stained with hematoxylin-eosin or Masson’s trichrome according to standard techniques.

### RNA extraction, RNA-seq analysis, and RT-qPCR analysis

Total RNA was extracted from the liver or cultured cells with the use of an RNeasy Mini Kit (Qiagen, Venlo, the Netherlands). Construction of RNA-sequencing (RNA-seq) libraries and sequencing with the Illumina HiSeq-PE150 platform were performed by Novogene (Beijing, China). Relative gene expression was evaluated on the basis of transcripts per million (TPM). For reverse transcription (RT) and quantitative polymerase chain reaction (qPCR) analysis, isolated RNA was subjected to RT with the use of Prime Script RT Master Mix (Takara Bio, Shiga, Japan) and the resulting cDNA was subjected to real-time PCR analysis with TB Green Premix Ex Taq II (Takara Bio) in a Thermal Cycler Dice Real Time System III (Takara Bio). Relative mRNA abundance was determined by the standard curve method, with normalization according to the amount of *Rplp0* mRNA. The sequences of the PCR primers are provided in Supplemental Table S1.

### Immunoblot analysis

Immunoblot analysis was performed as described previously^30^. In brief, cells were lysed in lysis buffer (20 mM Tris–HCl [pH 7.5], 150 mM NaCl, 2 mM EDTA, 1% Nonidet P-40, 10% glycerol) supplemented with protease and phosphatase inhibitors. The lysates were fractionated by SDS-polyacrylamide gel electrophoresis, and the separated proteins were transferred to a nitrocellulose membrane for immunoblot analysis with antibodies to total or phosphorylated (Ser^423/425^) forms of Smad3 (Cell Signaling Technology).

### Statistical analysis

Quantitative data are presented as means ± SEM and were compared between or among groups with the two-tailed unpaired Student’s *t* test or by analysis of variance (ANOVA) followed by Tukey’s post hoc test, respectively. A *p* value of < 0.05 was considered statistically significant.

## Results

### Pioglitazone treatment increases the amount of LA metabolites in adipose tissue of KK-Ay mice

**Figure 1:**
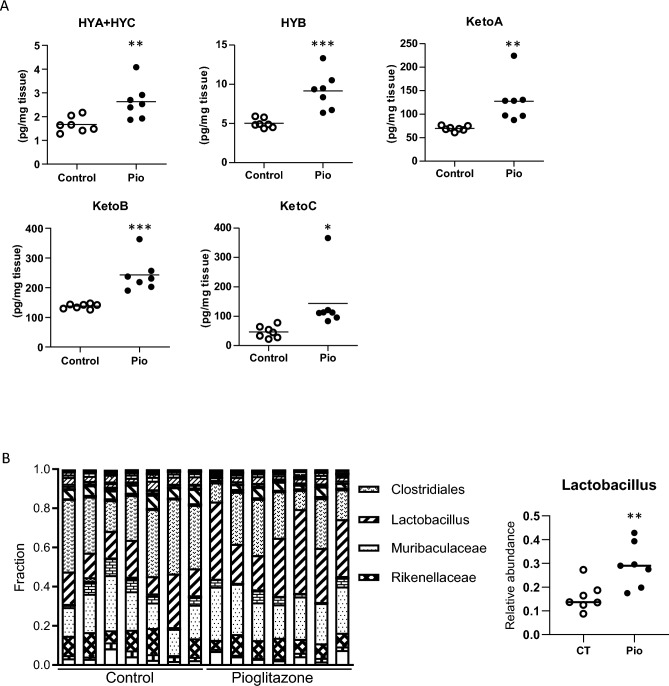
Effects of pioglitazone on the amount of LA metabolites in adipose tissue and on the gut microbiota in KK-Ay mice. (**A**) Concentrations of LA metabolites in epididymal adipose tissue of control mice or mice treated with pioglitazone (Pio) for 4 weeks. HYA + HYC; sum of the amounts of HYA and HYC. Data are shown for seven mice of each group, with the horizontal bar indicating the mean. *p < 0.05, **p < 0.01, ***p < 0.001 versus control mice (Student’s *t* test). (**B**) Gut microbiota profile at the genus level for feces of control mice or mice treated with pioglitazone for 3 weeks as determined by 16S rRNA gene analysis. Only bacteria that account for > 5% of the total are shown. Data are presented for seven mice of each group.

To clarify the mechanism underlying the amelioration of NAFLD/NASH by pioglitazone, we performed lipidomics analysis of adipose tissue, the target tissue of pioglitazone, in KK-Ay mice treated with this antidiabetic agent for 4 weeks. Compared with control mice, pioglitazone-treated mice showed significant increases in the abundance of gut microbial LA metabolites including HYA + HYC, 10-hydroxy-octadecanoic acid (HYB), 10-oxo-*cis*-12-octadecenoic acid (KetoA), 10-oxo-octadecanoic acid (KetoB), and 10-oxo-*trans*-11-octadecenoic acid (KetoC) (Fig. 1A). Given that these metabolites are produced from LA by lactic acid bacteria represented by *Lactobacillus* in the gut^28^, we hypothesized that pioglitazone might affect the gut microbiota. Analysis of 16S rRNA gene amplicons derived from feces revealed a substantial increase in the fraction of *Lactobacillus* in KK-Ay mice treated with pioglitazone for 3 weeks compared with control mice (Fig. 1B). These results thus suggested that pioglitazone treatment increases the gut microbial LA metabolite content in adipose tissue by influencing the gut microbiota.

### HYA ameliorates liver fibrosis in mice fed a high-fat diet

**Figure 2:**
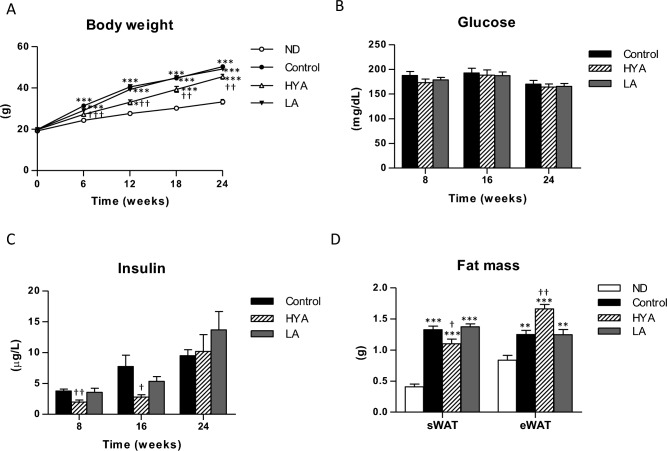
Effects of HYA on body weight, blood glucose and plasma insulin concentrations, and adipose tissue mass in mice fed a high-fat diet. (**A**) Time course of body weight in C57BL/6 J mice fed a normal diet (ND), a high-fat diet (control), or a high-fat diet containing 1% HYA or 1% LA. (**B** and **C**) Blood glucose (**B**) and plasma insulin (**C**) concentrations at 8, 16, or 24 weeks after treatment onset for mice as in (**A**). (**D**) Mass of subcutaneous adipose tissue (sWAT) and epididymal adipose tissue (eWAT) at 26 weeks after treatment onset for mice as in (**A**). All data are means ± SEM (ND; *n* = 6, control, HYA, and LA; *n* = 10 mice per group). *p < 0.05, **p < 0.01, ***p < 0.001 versus ND-fed mice; †*p* < 0.05, ††*p* < 0.01, †††*p* < 0.001 versus control mice (one-way ANOVA followed by Tukey’s test).

To study whether LA metabolites contribute to the amelioration of NAFLD/NASH by pioglitazone, we investigated the effects of HYA, a major gut microbial LA metabolite^28^, in C57BL/6 J mice fed a high-fat diet for a prolonged period. The gain in body weight was significantly suppressed in mice fed a high-fat diet supplemented with HYA compared with those fed the high-fat diet alone (control), whereas similar administration of LA had no such effect (Fig. 2A). Food intake did not differ between HYA- or LA-treated mice and control mice (Supplemental Figure S1A). Whereas blood glucose concentrations also did not differ among these three groups of mice, the plasma insulin concentration at 8 and 16 weeks after treatment onset was significantly lower in HYA-treated mice than in control mice (Fig. 2B and C), suggesting that HYA attenuates insulin resistance. The plasma concentrations of triglyceride, cholesterol, and NEFA did not differ among control and LA- or HYA-treated mice (Supplemental Figure S1B–D). The mass of subcutaneous adipose tissue was decreased and that of epididymal adipose tissue was slightly but significantly increased in HYA-treated mice compared with control mice at 26 weeks after treatment initiation (Fig. 2D).

**Figure 3:**
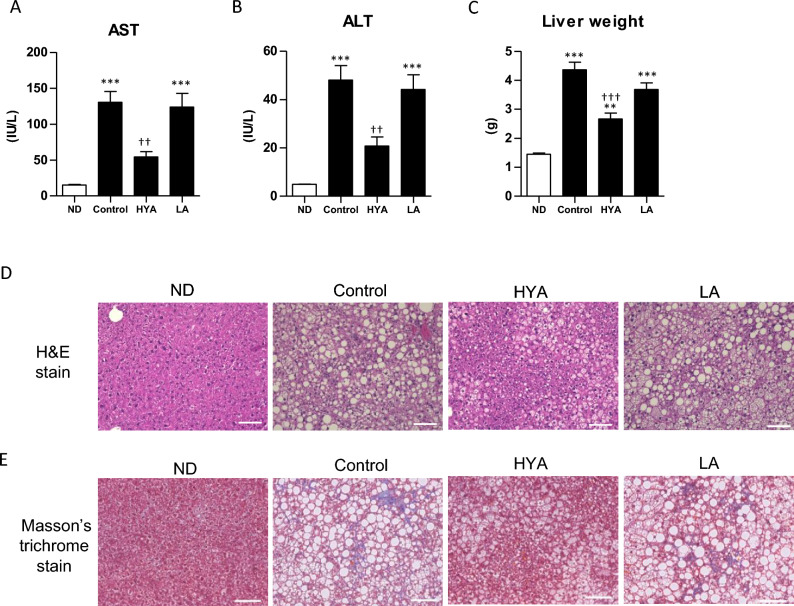
Effects of HYA treatment on liver histology in mice fed a high-fat diet. (**A** and **B**) Plasma levels of AST (**A**) and ALT (**B**) in C57BL/6 J mice fed a normal diet (ND), a high-fat diet (Control), or a high-fat diet containing 1% HYA or 1% LA for 24 weeks. (**C**) Liver weight of mice as in (**A**) after treatment for 26 weeks. Data in (**A**) through (**C**) are means ± SEM (ND; *n* = 6, control, HYA, and LA; *n* = 10 mice per group). **p < 0.01, ***p < 0.001 versus ND-fed mice; ††*p* < 0.01, †††*p* < 0.001 versus control mice (one-way ANOVA followed by Tukey’s test). (**D** and **E**) Representative staining of hepatic sections with hematoxylin-eosin (H&E) (**D**) or with Masson’s trichrome (**E**) for mice as in (**C**). Scale bars, 100 µm.

The increase in plasma levels of AST and ALT induced by the high-fat diet was significantly attenuated by HYA treatment for 24 weeks but was unaffected by LA administration (Fig. 3A and B). Liver weight was also suppressed in HYA-treated mice compared with control mice (Fig. 3C). Histological analysis revealed mild fibrosis as well as micro- and macrovesicular steatosis and inflammatory cell infiltration in the liver of control mice. HYA administration for 26 weeks markedly ameliorated these histological features of the liver, whereas LA administration had no such effect (Fig. 3D and E). The LA metabolite HYA thus specifically ameliorated hepatic histological changes induced by long-term feeding with a high-fat diet.

### HYA treatment suppresses the expression of fibrosis-related genes in the liver of mice fed a high-fat diet

**Figure 4:**
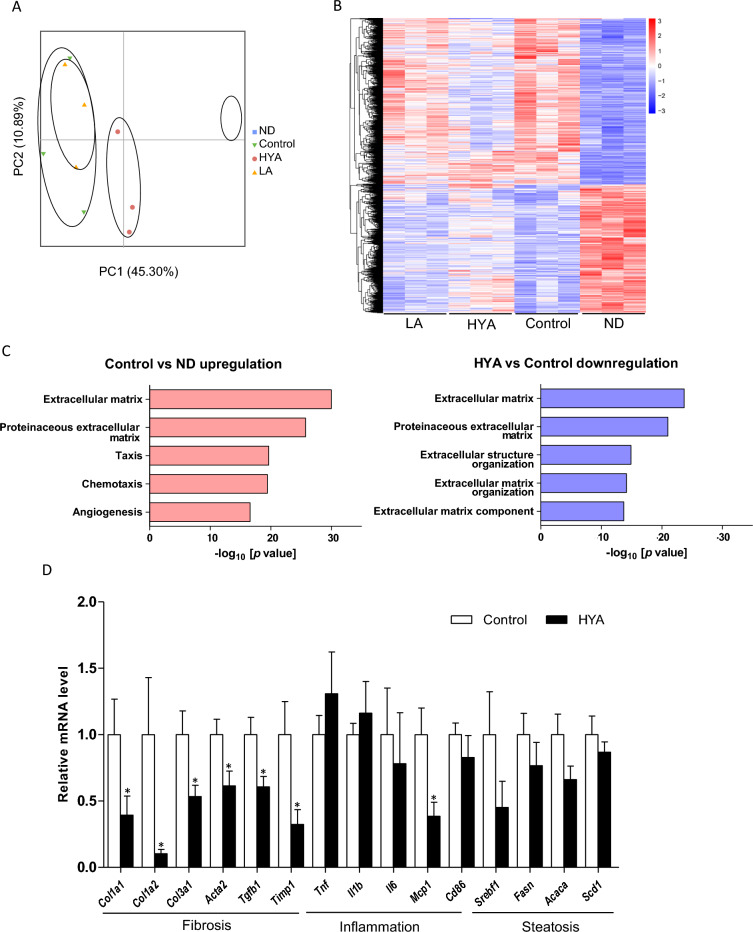
Effects of HYA on hepatic gene expression in mice fed a high-fat diet. (**A**) Principal component (PC) analysis of RNA-seq data for the liver of C57BL/6 J mice fed a normal diet (ND), a high-fat diet (control), or a high-fat diet containing 1% HYA or 1% LA for 26 weeks (*n* = 3 mice per group). (**B**) Heat map for hierarchical clustering of differentially expressed genes in the three independent samples per group in (**A**). (**C**) GO analysis of significantly upregulated genes (log_2_[fold change] of > 1, adjusted *p* value of < 0.05) in the liver of control versus ND-fed mice as well as of downregulated genes (log_2_[fold change] of <  − 1, adjusted *p* value of < 0.05) in the liver of HYA-treated mice versus control mice. (**D**) RT-qPCR analysis of mRNA abundance for the indicated genes in the liver of C57BL/6 J mice fed a high-fat diet (control) or a high-fat diet containing 1% HYA for 26 weeks. Data are means ± SEM (*n* = 6 mice per group). *p < 0.05 versus control mice (Student’s *t* test).

To elucidate the mechanism by which HYA improves hepatic histology in mice fed a high-fat diet, we investigated gene expression profiles of the liver by RNA-seq analysis. The pattern of gene expression differed greatly between mice fed a normal diet and those fed a high-fat diet (Fig. 4A and B). Whereas LA treatment did not affect the pattern of hepatic gene expression in high-fat diet–fed mice, HYA treatment altered such gene expression so that the pattern more closely resembled that of mice fed a normal diet (Fig. 4A and B). Gene ontology (GO) enrichment analysis of the RNA-seq data revealed that the top two categories were extracellular matrix (ECM) and proteinaceous ECM for genes upregulated by feeding a high-fat diet as well as for genes downregulated by HYA treatment (Fig. 4C). Excessive accumulation of ECM in the liver due to an imbalance between its production and degradation contributes to the pathogenesis of hepatic fibrosis^31,32^. Indeed, RT-qPCR analysis showed that mRNA abundance for genes related to fibrosis—such as those for collagen (*Col1a1*, *Col1a2*, and *Col3a1*), α–smooth muscle actin (*Acta2*), TGF-β1 (*Tgfb1*), and tissue inhibitor of metalloproteinase 1 (*Timp1*)—was markedly decreased in the liver of HYA-treated mice compared with that of control mice (Fig. 4D). Hepatic expression of genes related to steatosis tended to be decreased in HYA-treated mice compared with control mice, but the differences did not achieve statistical significance (Fig. 4D). With regard to inflammation-related genes, hepatic expression of the monocyte chemoattractant protein–1 gene (*Mcp1*) was significantly decreased and that of the interleukin-6 gene (*Il6*) tended to be decreased in HYA-treated mice compared with control mice (Fig. 4D). Together, these results suggested that HYA treatment ameliorates liver fibrosis induced by a high-fat diet.

### HYA suppresses activation of TGF-β–Smad3 signaling in hepatic stellate cells

**Figure 5:**
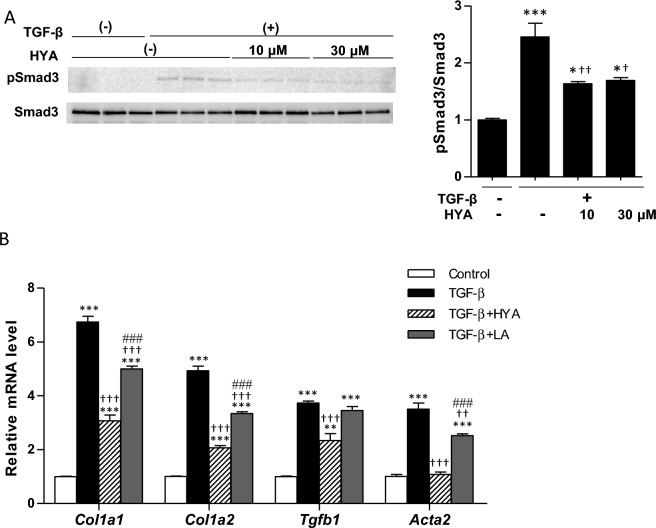
Effect of HYA on TGF-β signaling in human hepatic stellate LX-2 cells. (**A**) Immunoblot analysis of total and phosphorylated (p) forms of Smad3 in LX-2 cells incubated in the absence or presence of TGF-β (1 ng/mL) and HYA (10 or 30 µM) for 2 h. A representative blot and densitometric quantitation of the pSmad3/Smad3 band intensity ratio are shown. (**B**) RT-qPCR analysis of mRNA abundance for fibrosis-related genes in LX-2 cells incubated in the absence (control) or presence of TGF-β (1 ng/ml), HYA (10 μM), or LA (10 μM) for 24 h. All quantitative data are means ± SEM from three independent experiments (*n* = 3). *p < 0.05, **p < 0.01, ***p < 0.001 versus control; †*p* < 0.05, ††*p* < 0.01, †††*p* < 0.001 versus TGF-β alone; ###*p* < 0.001 versus TGF-β plus HYA (one-way ANOVA followed by Tukey’s test).

Hepatic fibrosis involves activation of hepatic stellate cells and consequent enhancement of ECM production^14^. TGF-β is a key fibrogenic factor that activates hepatic stellate cells and promotes fibrosis through a Smad3-dependent pathway^15^. To uncover the mechanism by which HYA ameliorates liver fibrosis, we examined its effect on TGF-β signaling and action in LX-2 human hepatic stellate cells. TGF-β induced the phosphorylation of Smad3 in these cells, and this effect was suppressed by HYA (Fig. 5A). In addition, HYA treatment attenuated the induction of fibrosis-related gene expression by TGF-β in LX-2 cells, whereas LA treatment had a lesser or no such effect (Fig. 5B). These results suggested that HYA ameliorates liver fibrosis through suppression of the TGF-β–Smad3 signaling pathway in hepatic stellate cells.

## Discussion

Several clinical studies have shown that pioglitazone improves liver histology in NAFLD/NASH^21–27^, but the mechanism of this effect has remained unknown. We have now shown that pioglitazone treatment increased the concentration of LA metabolites in adipose tissue of KK-Ay mice. This effect was accompanied by an increase in the fraction of *Lactobacillus* in the gut microbiota. Furthermore, administration of the LA metabolite HYA attenuated hepatic histological changes including fibrosis in C57BL/6 J mice fed a high-fat diet. Finally, HYA attenuated the induction of fibrosis-related gene expression through suppression of TGF-β signaling. Our results thus implicate the LA metabolite HYA in the mechanism by which pioglitazone ameliorates NAFLD/NASH.

HYA administration markedly improved liver histology including steatosis, inflammatory cell infiltration, and fibrosis in mice fed a high-fat diet. Whereas GO analysis of RNA-seq data showed that a high-fat diet induced substantial upregulation of the expression of ECM-related genes, consistent with the results of previous studies^33,34^, the expression of such genes was normalized by concomitant HYA administration. Given that excessive production of ECM by hepatic stellate cells is a central contributor to the pathogenesis of liver fibrosis^15^, HYA may attenuate the fibrotic process by targeting ECM-related gene expression. Indeed, we have now also shown that HYA suppresses fibrosis-related gene expression by inhibiting TGF-β–Smad3 signaling in hepatic stellate cells. Hepatic fibrosis is the strongest predictor of liver-related events and long-term mortality in individuals with NAFLD/NASH^13,35^. By targeting liver fibrosis, HYA may therefore be of therapeutic benefit for NAFLD/NASH patients.

In this study, HYA treatment did not alter blood glucose levels whereas this treatment ameliorated insulin resistance at an early stage of the treatment. The possible mechanism for this result may involve glucagon and incretin hormones. It has been reported that glucagon secretion by the long-chain fatty acids such as palmitic acid and DHA is suppressed in mice lacking GPR40 and GPR120^36^, suggesting that GPR40 and GPR120 mediate glucagon secretion by long-chain fatty acid. Recently, HYA has been shown to activate GPR40 and GPR120 in intestinal L cells to increase the secretion of the incretin GLP-1^37^. These findings suggest that HYA may affect glucagon secretion via activation of GPR40 and GPR120 in pancreatic ∝-cells. Thus, in addition to ameliorating insulin resistance, HYA may modulate blood glucose levels by affecting the secretion of glucagon and incretin hormones.

We found that HYA administration suppressed weight gain and ameliorated insulin resistance in mice fed a high-fat diet, consistent with the results of a recent study^37^. In our study, the weight of epididymal adipose tissue was slightly but significantly increased while that of subcutaneous adipose tissue was decreased by HYA administration, suggesting that the amelioration of insulin resistance by HYA may not simply be due to a reduction in fat mass. Such attenuation of insulin resistance in a manner independent of fat mass is reminiscent of metabolic healthy obesity, characterized by obesity in the absence of metabolic and cardiovascular complications^38^. Although the mechanisms responsible for the distinction between metabolically healthy and metabolically unhealthy obesity are not well understood, factors such as chronic inflammation and fibrosis in adipose tissue, changes in adipokine levels, and adipocyte size are thought to play a role^39^. Indeed, HYA administration was previously shown to reduce the circulating level of the proinflammatory adipokine MCP-1 and to attenuate chronic inflammation in adipose tissue of mice with diet-induced obesity^37^. Furthermore, the gut microbial LA metabolite KetoA was shown to have effects on adipose tissue including upregulation of adiponectin secretion^40^. Further study of the effects of the gut microbial LA metabolites on adipose tissue and their underlying mechanisms may therefore provide important insight into metabolically healthy or unhealthy obesity.

LA metabolites including HYA are produced by lactic acid bacteria represented by *Lactobacillus* in the gut^28^. Our present results suggest that pioglitazone increases the LA metabolite content of host tissue by increasing the fraction of *Lactobacillus* in the gut microbiota. However, there was some variability in the fraction of *Lactobacillus* for each mouse treated with pioglitazone. Although the mechanism for this variability among each mouse remained undetermined in this study, some factor that differs among each mouse might be required for the increase of *Lactobacillus* by pioglitazone. Adipose tissue is the major target organ of pioglitazone, given that PPARγ is predominantly expressed in adipocytes^41^. Pioglitazone ameliorates insulin resistance by various mechanisms. It thus increases the number of small adipocytes by promoting the differentiation of preadipocytes into mature adipocytes, reduces the number of hypertrophic adipocytes by inducing apoptosis, suppresses proinflammatory adipokine production, and increases the production of adiponectin^42–44^. PPARγ is also expressed in other cell types including colonic epithelial cells and immune cells^45,46^, although its expression level is relatively low in these cells compared with adipocytes. Consistent with this expression pattern, pioglitazone suppresses inflammation and improves intestinal barrier function in colitis^47,48^. It is thus possible that pioglitazone influences the gut microbiota through its effects on adipose tissue or, more directly, through those on intestinal epithelial cells and immune cells in the intestinal tract. Studies with tissue- or cell type–specific PPARγ knockout mice may shed light on which tissues or cells mediate the effect of pioglitazone on the gut microbiota.

In conclusion, our findings suggest that the gut microbial LA metabolite HYA plays a role in the mechanism by which pioglitazone ameliorates NAFLD/NASH. We have also revealed a previously unrecognized mechanism by which HYA suppresses fibrosis-related gene expression through inhibition of TGF-β–Smad3 signaling in hepatic stellate cells. Its ability to target liver fibrosis may render HYA of therapeutic benefit for individuals with NAFLD/NASH.

### Supplementary Information

Supplementary Information.

## Data Availability

Data, analytic methods, and study materials are all available on request to the corresponding author. Original RNA-seq data have been deposited in the Gene Expression Omnibus database of NCBI (GSE242881).
