# Supplementary material for: A gut microbial metabolite of linoleic acid ameliorates liver fibrosis by inhibiting TGF-β signaling in hepatic stellate cells
Source: Sci Rep. 2023 Nov 3;13:18983. doi: 10.1038/s41598-023-46404-5 (PMC10624680; doi:10.1038/s41598-023-46404-5)

Supplementary Information for

A gut microbial metabolite of linoleic acid ameliorates liver fibrosis by inhibiting TGF-β signaling in hepatic stellate cells

Nanaho Kasahara[1,8], Yukiko Imi[1,8], Reina Amano[1], Masakazu Shinohara[2,3], Kumiko Okada[4], Yusei Hosokawa[4], Makoto Imamori[4], Chiaki Tomimoto[5], Jun Kunisawa[6], Shigenobu Kishino[7], Jun Ogawa[7], Wataru Ogawa[4], Tetsuya Hosooka[1,4,6,*]

Corresponding author: Tetsuya Hosooka
Email: thosooka@u-shizuoka-ken.ac.jp

This PDF file includes:

- Supplementary Table S1
- Supplementary Figures S1
- Full length images of western blotting

Supplemental Table S1

Sequence of primers for RT-qPCR analysis.
Sequence of primers for the indicated genes for RT-qPCR analysis is shown.

Species	Gene	Forward primer (5' → 3')	Reverse primer (5' → 3')
Mouse	*Col1a1*	CCTCAGGGTATTGCTGGACAAC	ACCACTTGATCCAGAAGGACCTT
	Col1a2	GTAACTTCGTGCCTAGCAACA	CCTTTGTCAGAATACTGAGCAGC
	Col3a1	CTGTAACATGGAAACTGGGGAAA	CCATAGCTGAACTGAAAACCACC
	Acta2	GTCCCAGACATCAGGGAGTAA	TCGGATACTTCAGCGTCAGGA
	Tgfb1	CTCCCGTGGCTTCTAGTGC	GCCTTAGTTTGGACAGGATCTG
	Timp1	GCAACTCGGACCTGGTCATAA	CGGCCCGTGATGAGAAACT
	Tnf	GGTCCCCAAAGGGATGAG	TCCTCCACTTGGTGGTTTG
	Il1b	TTCAGGCAGGCAGTATCACTC	GAAGGTCCACGGGAAAGACAC
	Il6	TAGTCCTTCCTACCCCAATTTCC	TTGGTCCTTAGCCACTCCTTC
	Mcp1	CCAACTCTCACTGAAGCCAGCTC	TTGGGATCATCTTGCTGGTGAA
	Cd86	TGTTTCCGTGGAGACGCAAG	TTGAGCCTTTGTAAATGGGCA
	Srebf1	AGCCATGGATTGCACATTT	CACGGACGGGTACATCT
	Fasn	GCTGGCATTCGTGATGGAGTCGT	AGGCCACCAGTGATGATGTAACTCT
	Acaca	GATGAACCATCTCCGTTGGC	GACCCAATTATGAATCGGGAGTG
	Scd1	TTCTTGCGATACACTCTGGTGC	CGGGATTGAATGTTCTTGTCGT
	Rplp0	GAGGAATCAGATGAGGATATGGGA	AAGCAGGCTGACTTGGTTGC
Human	*Col1a1*	GAACGCGTGTCATCCCTTGT	GAACGAGGTAGTCTTTCAGCAACA
	Col1a2	TCAAACTGGCTGCCAGCAT	CAAGAAACACGTCTGGCTAGG
	Tgfb1	ACCTGAACCCGTGTTGCTCT	CTAAGGCGAAAGCCCTCAAT
	a-Sma	CCGACCGAATGCAGAAGG	ACAGAGTATTTGCGCTCCGGA
	Rplp0	ATGCAGCAGATCCGCATGT	TTGCGCATCATGGTGTTCTT

Supplementary Figure S1

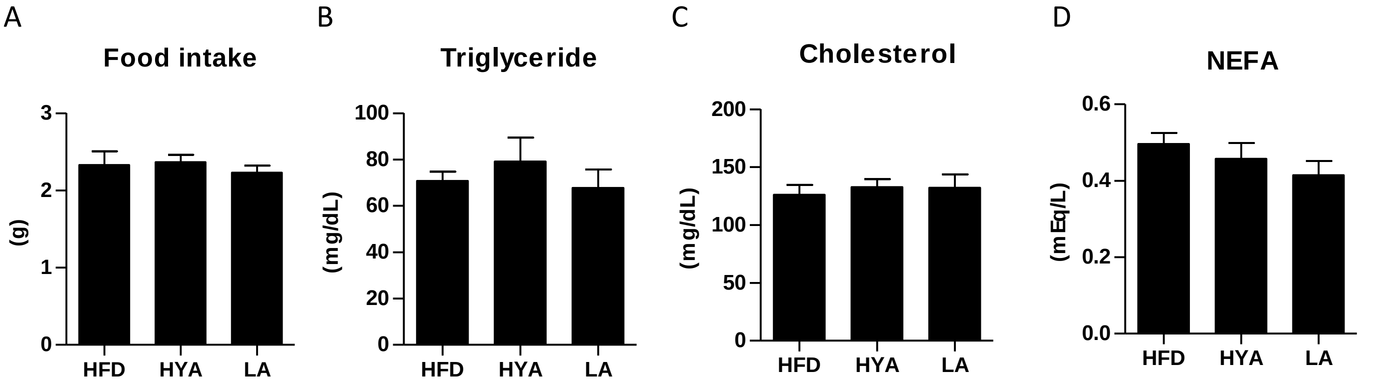

Supplementary Figure S1: Lack of effect of HYA treatment on food intake and plasma triglyceride, cholesterol, and NEFA levels in mice fed a high-fat diet.

(A) Food intake at 4 to 5 weeks after the onset of feeding C57BL/6J mice with a high-fat diet (HFD) supplemented or not with 1% HYA or 1% LA. (B-D) Plasma triglyceride (B), total cholesterol (C), and NEFA (D) levels in mice as in (A) at 24 weeks after the onset of HFD feeding. All data are means ± SEM (n = 6 to 10 mice per group).

Full length images of western blotting

Blot: pSmad3

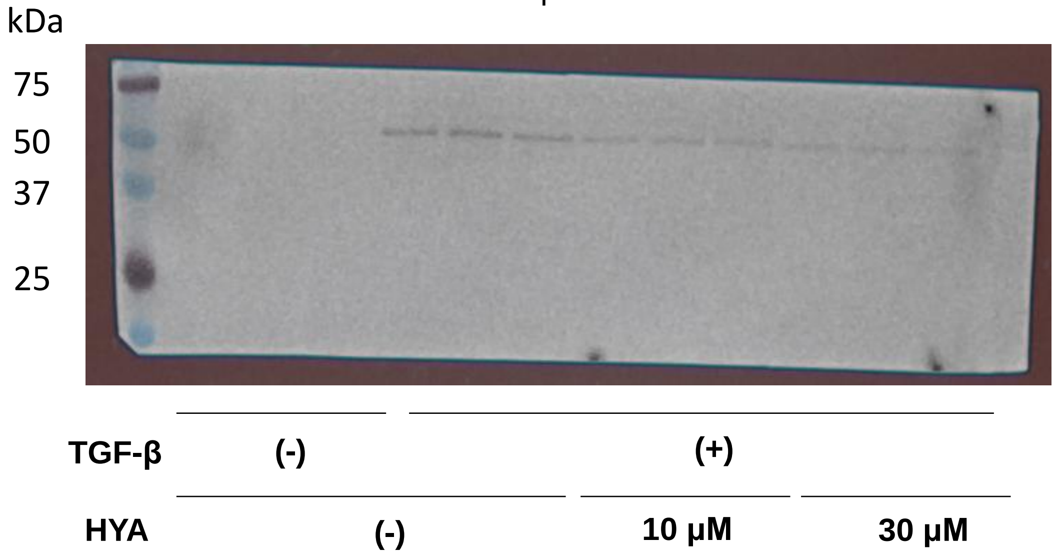

Blot: Smad3

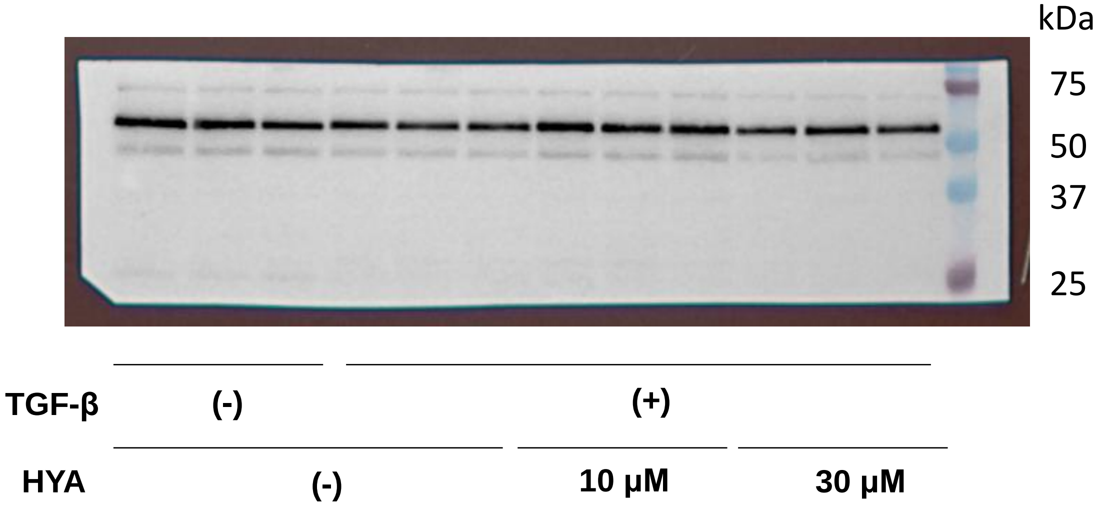

Supplement: Supplementary file 1 — Supplementary Information. [file 41598_2023_46404_MOESM1_ESM.pdf]
